# A Post-surgical Collection Is Not Just a Collection: A Case Report of Colorectal Cancer Metastasis

**DOI:** 10.7759/cureus.84406

**Published:** 2025-05-19

**Authors:** Anurag Agarwal, Sjaak Pouwels, Ahmed Ahmed, Suhaib Ahmad

**Affiliations:** 1 Department of General Surgery, Apollo Hospitals, Bangalore, IND; 2 Department of General Surgery, Betsi Cadwaladr University Health Board, Bangor, GBR; 3 Department of Intensive Care Medicine, Elisabeth-Tweesteden Hospital, Tilburg, NLD; 4 Department of Surgery, Bielefeld University-Campus Lippe, Detmold, DEU; 5 Department of General Surgery, Imperial College London, London, GBR; 6 Department of General Surgery, Ysbyty Gwynedd Hospital, Bangor, GBR; 7 Department of Emergency Medicine, Inselspital University Hospital of Bern, Bern, CHE

**Keywords:** colon cancer recurrence, colon cancer survillence, colo-rectal cancer, gastro-intestinal surgery, metastatic colo-rectal cancer

## Abstract

Subdiaphragmatic metastases in colorectal cancer are rare and can present as persistent intra-abdominal collections post-surgery. An elderly male in his mid-70s with colorectal adenocarcinoma (pT4a pN1c R0) developed a chronic subdiaphragmatic collection after undergoing an emergency right hemicolectomy for an obstructing tumor. Initially presumed to be a benign post-surgical complication, the collection persisted despite standard management. Subsequent investigation revealed metastatic adenocarcinoma, highlighting an uncommon metastatic pattern. This case highlights the need for a high index of suspicion in post-surgical patients when conventional explanations fail to explain clinical progression. Early consideration of metastatic disease and timely tissue sampling can significantly impact management and prognosis. This case reinforces the importance of clinical vigilance, advanced imaging, and multidisciplinary collaboration in recognizing rare metastatic patterns and optimizing treatment strategies in complex oncologic care.

## Introduction

Colorectal cancer (CRC) is a leading cause of morbidity and mortality worldwide, with surgical resection being the primary curative treatment [1]. However, post-operative complications, including intra-abdominal collections, can pose diagnostic and management challenges. Subdiaphragmatic collections are uncommon post-hemicolectomy, and their persistence often raises concerns about infection, anastomotic leaks, or malignancy [2,3].

Metastatic colorectal cancer (mCRC) is a leading cause of cancer-related mortality, with the liver and lungs being the most common metastatic sites due to portal venous drainage patterns. However, atypical metastasis can occur, influenced by factors such as tumor biology, molecular alterations (e.g., KRAS and BRAF mutations), and prior treatments altering metastatic pathways [4]. Unusual sites include the brain, bone, and peritoneum, often linked to advanced disease or hematogenous spread [5]. This case presents a rare manifestation of metastatic adenocarcinoma mimicking a chronic subdiaphragmatic collection, emphasizing the need for a meticulous diagnostic approach to uncover the underlying pathology and optimize therapeutic strategies.

## Case presentation

An elderly male in his mid-70s has a significant past medical history of hyperthyroidism, atrial fibrillation, osteoarthritis, irritable bowel syndrome (IBS), and psoriasis.

Initial presentation and surgery

He was diagnosed with CRC following admission to the Emergency Department for large bowel obstruction, which led to bowel perforation. He underwent an emergency right hemicolectomy, with histopathology confirming a pT4a pN1c R0, moderately differentiated adenocarcinoma of the ascending colon with a KRAS mutation but no NRAS and BRAF mutation, no dihydropyrimidine dehydrogenase (DPYD) variance, no microsatellite instability (MSI), and no neurotrophic tyrosine receptor kinase (NTRK). Post-operatively, the Colorectal Multidisciplinary Team (MDT) conducted an assessment, which recommended considering adjuvant chemotherapy. The patient was then referred to the oncology team for further evaluation. However, due to his poor performance status, the patient was considered unfit for adjuvant chemotherapy.

Postoperative surveillance and recurrence

**Figure 1:**
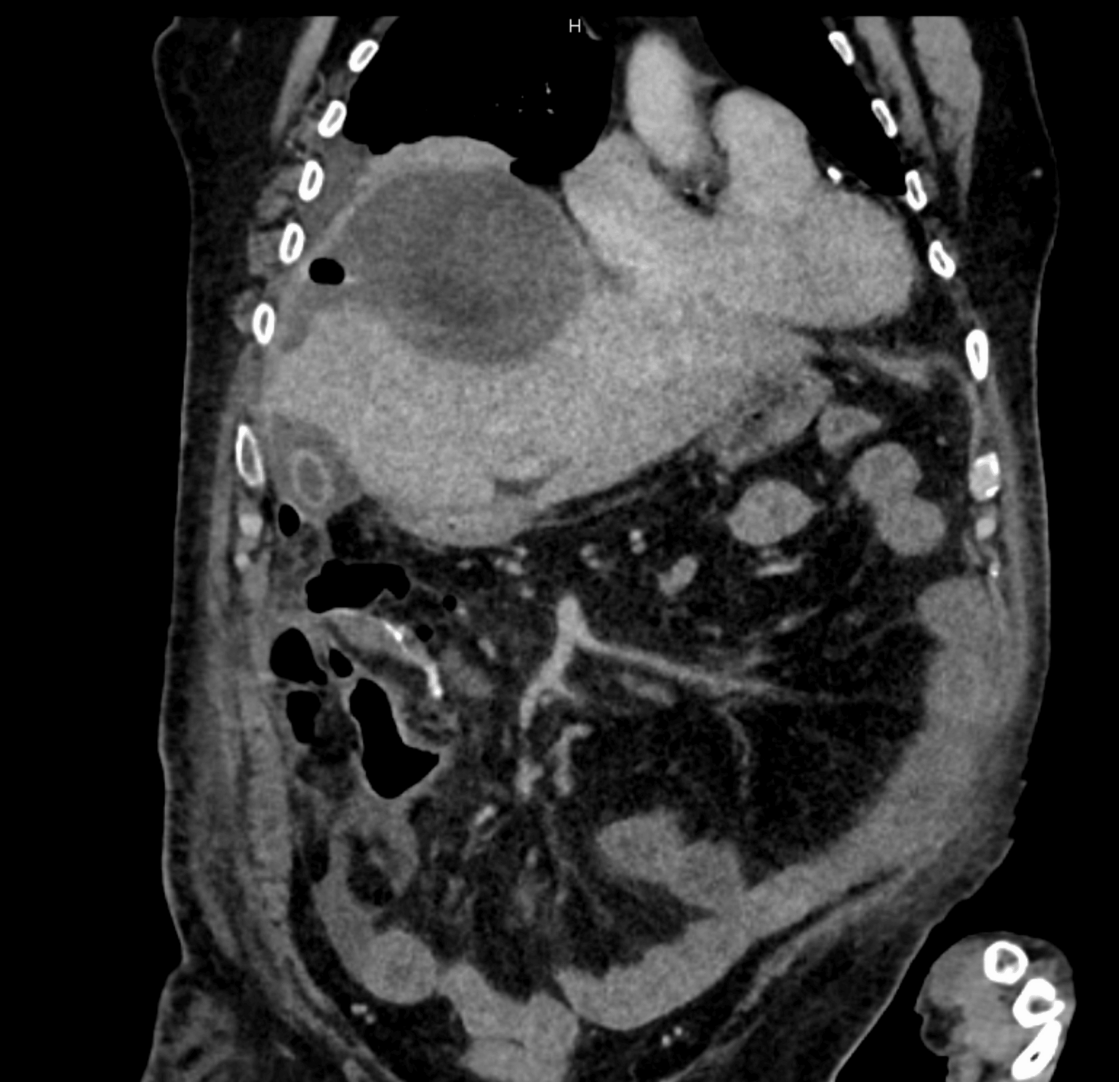
Post-operative Day 9, CT abdomen and pelvis showing a subdiaphragmatic collection. CT: computed tomography.

**Figure 2:**
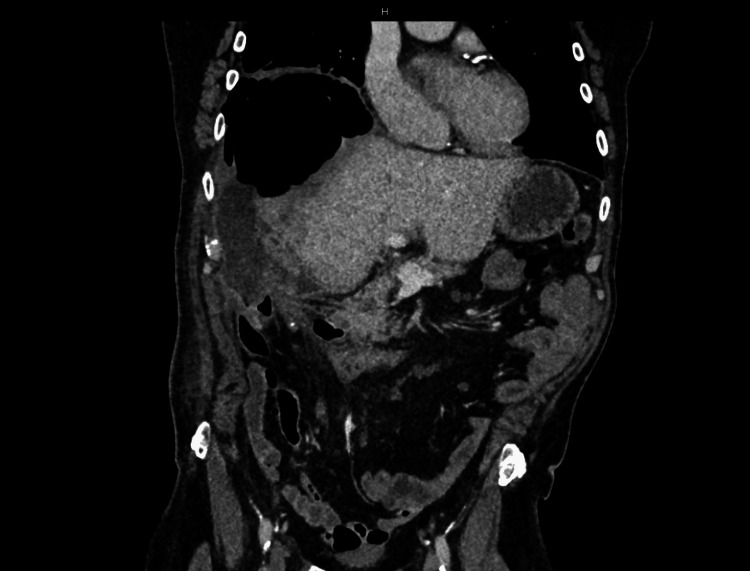
Surveillance CT of the abdomen and pelvis one year post-surgery, showing a subdiaphragmatic collection containing gas pockets. CT: computed tomography.

Subsequent imaging revealed a right subdiaphragmatic collection, initially thought to be a benign post-surgical sequela, as shown in Figure 1. The patient remained under surveillance, with serial imaging showing stability of the collection, no signs of recurrence or metastatic disease, and unremarkable tumor markers. Nine months later, he presented with sepsis and was admitted under the care of the on-call surgical team. A CT scan showed a persistent subdiaphragmatic collection, which had now increased in size and become gas-filled, as shown in Figure 2. He was then reassessed by the Colorectal MDT and was planned for Interventional Radiology (IR) to drain the collection, with the option of surgical management if IR drainage was unsuccessful. Due to its chronicity and the lack of definitive findings, IR declined drainage at that time, and he was managed conservatively with antibiotics.

Diagnostic re-evaluation and management

**Figure 3:**
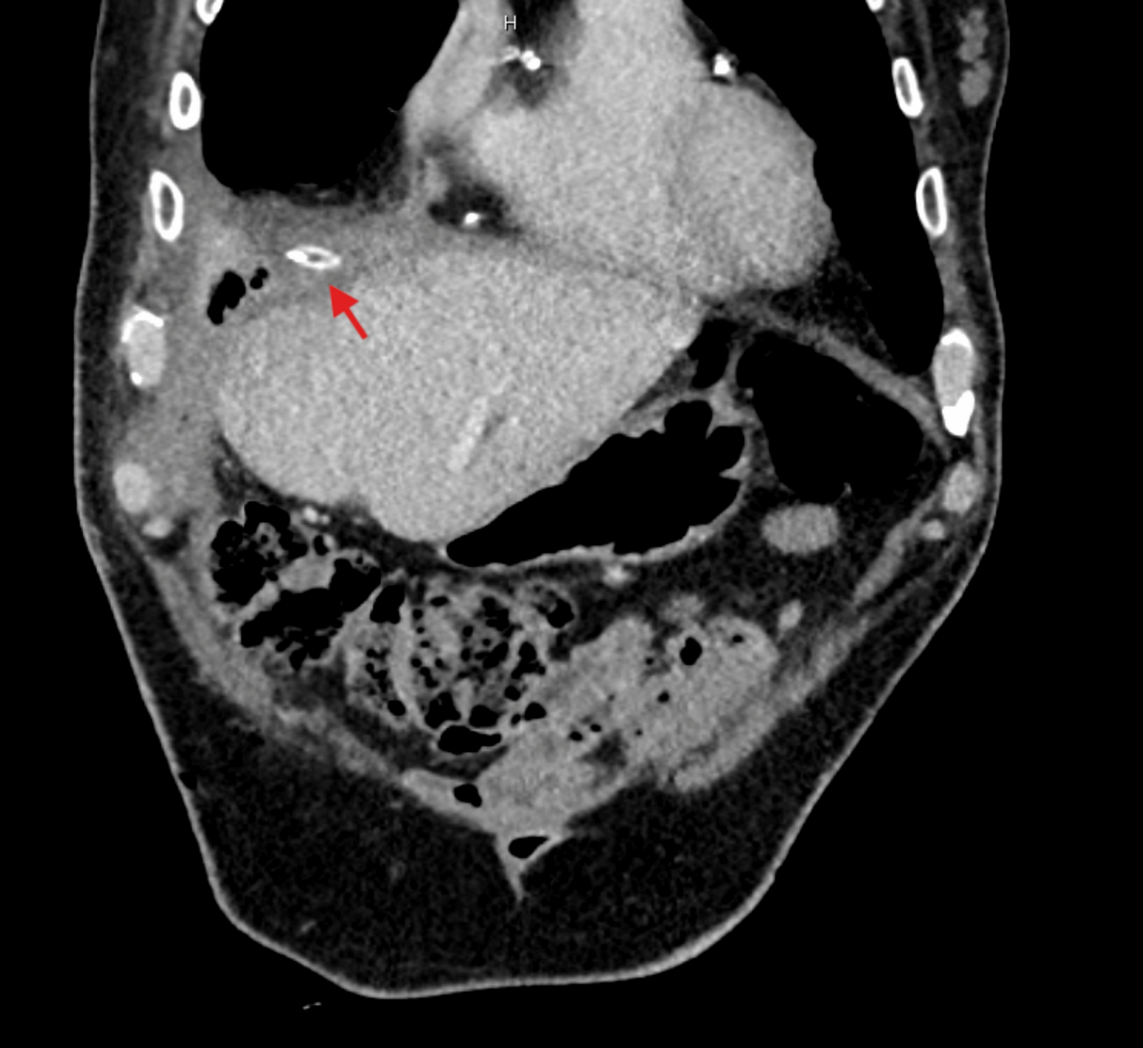
Post-operative CT of the abdomen and pelvis showing a surgical drain (marked with a red arrow), placed in anterior aspect of the liver within the subdiaphragmatic collection. CT: computed tomography.

Despite clinical improvement and subsequent discharge, further surveillance imaging showed persistence of the collection. Due to the unusual nature of the findings, the case was re-evaluated by the Colorectal MDT, which recommended further assessment. The patient underwent a repeat CT scan, which showed progression in the size and complexity of the collection, likely a fistula between the duodenum and the collection. This prompted the decision to proceed with drainage of the collection, which was performed surgically, and a drain was left in situ, as shown in Figure 3.

Cytological analysis of the drained fluid showed that the cellular specimen predominantly comprises acute inflammatory cells, with the accompanying cell block revealing scattered groups of atypical cells arranged in an acinar pattern. These cells display large vesicular nuclei, prominent nucleoli, and are embedded within a mucinous background. Immunohistochemical profiling demonstrates strong expression of CK20, CDX2, SATB2, and CEA, while CK7 and calretinin are notably absent. Taken together, these morphological and immunophenotypic features are diagnostic of metastatic adenocarcinoma of colorectal origin. Given these findings, the MDT recommended a defunctioning stoma to prevent further contamination and reduce the risk of complications, as well as, for palliative chemotherapy. The patient subsequently underwent a loop ileostomy, which was uneventful, and he recovered well post-operatively.

Differential Diagnosis

The differential diagnosis for the right-sided subdiaphragmatic collection included a post-operative seroma or abscess, a biliary or duodenal fistula, an infective subdiaphragmatic abscess, and recurrent colorectal carcinoma with associated peritoneal metastases. Each possibility was carefully considered in the context of the patient's clinical history, imaging characteristics, and evolving biochemical markers.

Treatment

The patient was initially managed conservatively with serial imaging and administration of intravenous antibiotics during episodes of sepsis. Owing to progression of the subdiaphragmatic collection, image-guided percutaneous drainage was subsequently performed, with histopathological analysis of the aspirated material confirming metastatic adenocarcinoma of colorectal origin. In view of the ongoing risk of fecal contamination and recurrent abscess formation, a loop ileostomy was subsequently fashioned. Following surgical intervention, the patient was referred to oncology services for assessment and initiation of palliative systemic chemotherapy.

Outcome and Follow-Up

Following ileostomy formation, the patient had two stoma reviews, reporting variable stool consistency with ongoing leakages. Examination revealed peristomal skin excoriation, which was managed with barrier wipes and convex pouch application. Nutritional support was emphasized due to ongoing weight loss, and a referral to a community dietitian was made.

The patient was keen to explore further surgical options before commencing chemotherapy. However, after further MDT discussion, palliative chemotherapy was considered the most appropriate course. He was reviewed by the oncology team and, due to his poor frailty and poor performance score, was placed on community-based supportive care. He eventually passed away in the hospital due to acute kidney injury (AKI) secondary to sepsis and disease progression.

## Discussion

Subdiaphragmatic metastases from CRC are rare and often represent an atypical metastatic pattern. These metastases are typically identified post-surgically, especially after procedures like colectomy or liver resection [6]. The metastatic spread to this area may be via direct extension, hematogenous routes, or due to peritoneal dissemination. Following perforation, it is plausible that tumor cells disseminate via the flow of peritoneal fluid, with subsequent implantation in dependent areas such as the subdiaphragmatic space. The dynamics of peritoneal fluid circulation, governed by both gravitational forces and the negative intrathoracic pressure generated during respiration, facilitate the movement of free-floating malignant cells toward regions of fluid reabsorption, including the omentum and the undersurfaces of the diaphragm, particularly the right hemidiaphragm [7]. Ascitic fluid tends to accumulate in the pelvic cavity and paracolic gutters, from where it is directed superiorly toward the diaphragm, promoting transcoelomic spread. This physiologic pathway may contribute to the preferential deposition of tumor cells in the pelvis and subdiaphragmatic regions [8]. When suspected, imaging techniques such as CT or MRI, along with histological confirmation, are essential for accurate diagnosis [4,5].

Distinguishing chronic post-operative collections from malignant recurrence presents a significant diagnostic challenge. Subdiaphragmatic collections are often attributed to infection or surgical complications, with malignancy being a rare initial consideration [9]. However, given the persistent and evolving nature of the collection, further evaluation was warranted. One of the key learning points is the role of MDT discussions in guiding complex oncological decisions. This case highlights the importance of MDT in post-surgical collections, particularly when standard interventions prove inconclusive.

The initial rejection of radiological interventions underscores the importance of persistence in clinical decision-making when findings do not fit the expected pattern. The eventual histological confirmation of metastatic adenocarcinoma underscores the necessity of considering malignancy in atypical collections, even in long-standing post-surgical cases. The delay in recognizing metastatic spread underscores the need for early, repeated imaging and biopsy in atypical presentations. The decision to proceed with surgical intervention despite uncertain radiological findings was pivotal in achieving a definitive diagnosis.

This case highlights the need for heightened clinical vigilance regarding persistent intra-abdominal collections in patients with CRC. Given the rarity of subdiaphragmatic metastases in CRC, this case supports the hypothesis that unexplained collections should warrant earlier re-biopsy. Re-biopsy offers critical histopathological information, facilitating early detection of metastatic disease that might otherwise be overlooked. While routine surveillance typically monitors for recurrence, this case emphasizes the potential for missed diagnoses in the absence of re-biopsy. Early intervention, driven by more aggressive diagnostic strategies, can significantly alter patient management and outcome. Therefore, incorporating re-biopsy as part of the diagnostic workup in CRC patients with persistent abdominal collections could improve the detection of atypical metastatic patterns and guide more effective therapeutic decisions. Additionally, this case highlights the importance of holistic patient care. The patient’s nutritional status was compromised, necessitating interdisciplinary coordination to optimize his overall well-being.

Patient perspective

The patient and his family initially expressed concerns over the delay in recognizing the metastatic nature of his collection. However, upon discussion, they understood that his initial poor prognosis at the time of diagnosis meant that the ultimate treatment course would not have significantly differed. The patient appreciated the efforts to expedite his treatment and avoid unnecessary emergency department visits. During the patient’s hospitalization, his dentures were misplaced, significantly affecting his ability to maintain adequate nutritional intake. Recognizing the importance of this issue, multiple inquiries were made to locate the lost dentures. In the absence of their retrieval, arrangements were made for the patient to see a dentist upon discharge, with the costs for replacement covered by the responsible ward. Unfortunately, before these arrangements could be completed, the patient passed away.

## Conclusions

This case highlights the need for sustained clinical vigilance in post-surgical oncology. Persistent or atypical intra-abdominal collections must prompt reassessment, with malignancy considered, particularly in patients with a history of CRC. Multidisciplinary team involvement is crucial for managing complex cases, and holistic care addressing nutritional, psychosocial, and emotional needs remains essential to optimize outcomes. Strong patient advocacy is necessary to ensure timely investigation and to resolve administrative barriers that might delay critical interventions.
